# Performances of disseminated intravascular coagulation scoring systems in septic shock patients

**DOI:** 10.1186/s13613-020-00704-5

**Published:** 2020-07-10

**Authors:** Julie Helms, François Severac, Hamid Merdji, Raphaël Clere-Jehl, Bruno François, Emmanuelle Mercier, Jean-Pierre Quenot, Ferhat Meziani, for the CRICS TRIGGERSEP Group (Clinical Research in Intensive Care and Sepsis Trial Group for Global Evaluation and Research in Sepsis)

**Affiliations:** 1 Hôpitaux Universitaires de Strasbourg, Service de Médecine Intensive Réanimation, Nouvel Hôpital Civil, 1, Place de l’Hôpital, 67091 Strasbourg Cedex, France; 2 ImmunoRhumatologie Moléculaire, INSERM UMR_S1109, LabEx TRANSPLANTEX, Centre de Recherche d’Immunologie et d’Hématologie, Faculté de Médecine, Fédération Hospitalo-Universitaire (FHU) OMICARE, Fédération de Médecine Translationnelle de Strasbourg (FMTS), Université de Strasbourg (UNISTRA), Strasbourg, France; 3 Groupe Méthode en Recherche Clinique, Service de Santé Publique, Hôpitaux Universitaires de Strasbourg, Strasbourg, France; 4 Laboratoire de Biostatistique et d’Informatique Médicale, ICube, UMR 7357, Faculté de Médecine, Hôpitaux Universitaires de Strasbourg, Strasbourg, France; 5 INSERM (French National Institute of Health and Medical Research), UMR 1260, Regenerative Nanomedicine (RNM), FMTS, Strasbourg, France; 6 Inserm CIC1435 & UMR1092, CHU Dupuytren, Limoges, France; 7 Service de Réanimation Polyvalente, CHU Dupuytren, Limoges, France; 8 Service de Médecine Intensive Réanimation, Centre Hospitalier Universitaire Bretonneau, CRICS-TRIGGERSEP Network, Tours, France; 9 Service de Médecine Intensive-Réanimation, CHU Dijon Bourgogne, Dijon, France; 10 INSERM, U1231, Equipe Lipness, LipSTIC LabEx, Dijon, France; 11 INSERM, CIC 1432, Module Epidémiologie Clinique, Dijon, France

**Keywords:** Coagulopathy, Disseminated intravascular coagulation, DIC, Sepsis, Septic shock

## Abstract

**Background:**

There is no gold standard to diagnose septic shock-induced disseminated intravascular coagulation (DIC). The objective of our multicenter prospective study was to assess the performances of the different major scoring systems in terms of mortality prediction and DIC diagnosis. The JAAM-DIC 2016 score, the ISTH overt-DIC 2001 score, the associations of sepsis-induced coagulopathy (SIC) score with JAAM-DIC 2016 or ISTH overt-DIC scores were tested in patients within 12 h of their admission in ICU for septic shock (day 1) and at day 2.

**Results:**

582 patients were enrolled in the study. 182/567 (32.1%) were diagnosed with DIC according to ISTH overt-DIC score, and 193/561 (34.4%) according to JAAM-DIC score; 486/577 patients (84.2%) were diagnosed with a coagulopathy according to SIC score. A moderate concordance was observed between ISTH overt-DIC and JAAM-DIC [κ = 0.67 (0.60, 0.73), *p* < 0.001]. The delay of positivity of the scores for early DIC patients was not different between JAAM-DIC and ISTH overt-DIC scores. Although it was positive earlier, SIC score had worse diagnosis specificity, as 84.2% of the patients with septic shock were diagnosed with “coagulopathy”. The specificity of SIC score alone to predict mortality was very low [0.18 (0.15; 0.22)], compared to the ones of JAAM-DIC score [0.71 (0.67; 0.75)], and of ISTH overt-DIC score [0.76 (0.72; 0.80)], *p* < 0.001. The sensitivity of SIC score to predict mortality was 0.95 [0.89; 0.98], and the ones of JAAM-DIC score and ISTH overt-DIC score were 0.61 [0.50; 0.70] and 0.68 [0.58; 0.77], respectively. There was no benefit in sensitivity and specificity in combining SIC score to JAAM-DIC score or to ISTH overt-DIC score, compared to JAAM-DIC score or ISTH overt-DIC score alone.

**Conclusions:**

Our data suggest that the added value of SIC score alone or combined with other scores is limited, and that both JAAM-DIC score and ISTH overt-DIC score can be used in septic shock patients.

*Trial registration* clinicaltrial; Trial registration number: NCT02391792; Date of registration: 18/03/2015; URL of trial registry record: https://clinicaltrials.gov/ct2/show/NCT02391792?term=meziani&draw=4&rank=1

## Introduction

Since the currently available disseminated intravascular coagulation (DIC) scoring systems are not easy to use at bedside and physicians are unfamiliar with these scores, Iba et al. recently published recommendations for diagnosis of sepsis-induced DIC [1]. In septic patients with thrombocytopenia, they indeed recommended the use of a two-step process with simple diagnostic criteria for sepsis-induced coagulopathy (prothrombin time, PT/International Normalized Ratio and platelets) plus a Sequential Organ Failure Assessment (SOFA) score (which is the sum of four items: respiratory SOFA, cardiovascular SOFA, hepatic SOFA, renal SOFA), called sepsis-induced coagulopathy (SIC) score, eventually followed by the International Society on Thrombosis and Haemostasis (ISTH) overt-DIC score if SIC score is positive.

There is indeed no gold standard score to clearly define what is DIC and how to diagnose it in septic shock patients, sometimes leading to DIC being referred as “Disseminated International Confusion” [2]. Several scoring systems may be used by critical care physicians, but they all suffer from imperfections. First, the available scores fail to diagnose early asymptomatic DIC, also called “pre-DIC” or “non-overt” stages (coagulation system is activated but compensated); these patients are, however, as severe as those diagnosed with a patent DIC on intensive care unit (ICU) admission, with the same mortality rate [3]. Then, these scores fail to discriminate between patients with a prothrombotic activation of the coagulation and those with hemorrhagic and fibrinolytic states. Treatment of DIC patients probably depends on its stage (thrombotic, hemorrhagic, fibrinolytic), therefore underlying the importance of a prior proper stratification of the patients. Third, the diagnosis performances of the scores are unequal depending on the underlying disease, like is the presentation of DIC in the different clinical conditions (e.g., thrombotic DIC induced by sepsis versus bleeding DIC induced by hematologic malignancies). Different DIC scores or diagnosis markers might therefore be necessary for diagnosis, depending on the pathophysiological underlying condition [4]. Finally, repeating multiple coagulation tests to screen patients is costly [5].

When comparing the main scoring systems, the Japanese Association for Acute Medicine JAAM-DIC criteria were suggested to be more sensitive than the ISTH ones and allow an earlier diagnosis compared to the ISTH score [5]. Consequently, the JAAM-DIC score is preferred for early DIC-specific anticoagulant treatment assignment [6]. Interestingly for sepsis, the JAAM-DIC score does not use the fibrinogen decrease as a criterion, and it takes into account the kinetics of platelet decrease. The JAAM-DIC score has been revised in 2016, in order to align the new definition of sepsis and replace systemic inflammatory response syndrome (SIRS) criterion by antithrombin rate [7]. In spite of these apparent advantages over ISTH overt-DIC score, the JAAM score has been evicted from the last recommendations on DIC diagnosis [1].

We have therefore compared the diagnostic properties and performances in terms of mortality prediction of the different principal scoring systems (the revised version of JAAM-DIC 2016 score, ISTH overt-DIC 2001 score, the association of SIC and JAAM-DIC 2016 scores, and the association SIC and ISTH overt-DIC 2001 scores) in a multicenter prospective cohort of patients admitted on ICU for septic shock.

## Patients and methods

### Patients

Five hundred eighty-two consecutive adults (18 to 85 years old) referred for septic shock [8] and treated with norepinephrine and/or epinephrine, were prospectively enrolled after admission on ICU from four tertiary hospitals between 2015 and 2019. End-stage cardiac insufficiency (NYHA class IV), liver cirrhosis (Child–Pugh classification C) or ongoing cancers were excluded. The Strasbourg University Hospital Ethic Committee approved this multicentre study (NCT02391792) and it was registered on clinicaltrial.gov. Informed consent was obtained from the patient or relatives at admission, and confirmed by the patient when possible. Patients were managed following current guidelines without specific therapeutic intervention.

### Data sampling and evaluation of patients

Blood samples for global coagulation tests (platelet counts, prothrombin time (PT), fibrinogen, antithrombin and D-dimers) were collected within 12 h after the patients were admitted in the ICU (day 1), as well as on day 2.

All patients were followed up for 28 days after enrolment in the study, and 7- and 28-day all-cause mortality was assessed. Daily Sequential Organ Failure Assessment (SOFA) score was also documented.

### DIC and coagulopathy scoring systems (see Additional file 1: Table S1)

The JAAM-DIC 2016 score [7], the ISTH overt-DIC score [9], the association of SIC and JAAM-DIC 2016 scores, and the association of SIC and ISTH overt-DIC scores were calculated at days 1 and 2. Scores were considered positive as validated in original publications, if ISTH overt-DIC was 5 points or more, JAAM-DIC score was 4 points or more, and SIC was 4 points or more. The delay of positivity of the scores for DIC patients is the time between ICU admission for septic shock and the diagnosis of DIC according to the different scores.

### Baseline characteristics of the patients

Five hundred eighty-two consecutive patients were enrolled in the study. Median age was 69 [60; 77] years old and the male-to-female ratio was 377/205. Median simplified acute physiology score (SAPS) II score was 59 [48; 74] and mortality rate was 19.9% at day 7 and 35.5% at day 28. Patient characteristics during ICU stay, type of infection and bacteria are summarized in Table 1.

**Table 1:** Patient characteristics during ICU stay and mortality rate depending on coagulopathy scoring system

	All patients (*n* = 582)	Positive SIC score (*n* = 486/577)	Positive JAAM-DIC score (*n* = 193/561)	Positive ISTH overt-DIC score (*n* = 182/567)
D7-mortality, n (%)	115 (19.8)	105 (21.6)	60 (31.1)	69 (37.9)
D28-mortality, n (%)	207 (35.5)	181 (37.2)	95 (49.1)	107 (58.7)
SOFA (mean ± SD)	11.0 ± 3.2	11.2 ± 3.2	12.8 ± 2.9	12.8 ± 2.9
SAPS II (mean ± SD)	61.2 ± 19.3	64.1 ± 19.3	66.0 ± 19.2	67.8 ± 20.4
Mechanical ventilation, n (%)	518 (89.1)	437 (90.0)	180 (93.4)	172 (94.7)
Vasopressor, n (%)	582 (100)	486 (100)	193 (100)	182 (100)
Dialysis, n (%)	172 (29.5)	155 (31.9)	86 (44.5)	93 (51.1)
Nosocomial infection, n (%)	82 (14.1)	70 (14.4)	27 (14.0)	23 (12.6)
Infection source, n (%)				
Pneumonia	236 (40.5)	191 (39.3)	68 (35.2)	66 (36.3)
Urinary tract infection	102 (17.5)	89 (18.3)	43 (22.3)	38 (20.9)
Abdominal infection	89 (15.3)	74 (15.2)	36 (18.7)	34 (18.7)
Catheter infection	11 (1.9)	9 (1.9)	6 (3.1)	5 (2.7)
Positive blood culture^a^	136 (23.4)	114 (23.5)	53 (27.5)	52 (28.6)
Other	75 (12.9)	65 (13.4)	18 (9.3)	18 (9.9)
Bacteria, n (%)^b^				
Staphylococcus	99 (17.0)	74 (15.2)	33 (17.1)	26 (14.3)
Pneumococcus	41 (7.0)	35 (7.2)	15 (7.8)	14 (7.7)
Other GPC	68 (11.7)	54 (11.1)	21 (10.9)	25 (13.7)
*E. coli*	118 (20.3)	105 (21.6)	44 (22.8)	46 (25.3)
KES	90 (15.5)	78 (16.0)	28 (14.5)	28 (15.3)
Pseudomonas	33 (5.7)	30 (6.2)	12 (6.2)	12 (6.6)
Other GNB	84 (14.4)	69 (14.2)	27 (14.0)	27 (14.8)
Other	38 (6.5)	36 (7.4)	5 (2.6)	8 (4.4)

Scores were positive if ISTH overt-DIC score was ≥ 5 points, JAAM-DIC score was ≥ 4 points, and SIC was ≥ 4 points at day 1 or 2 after ICU admission for septic shock

*GNB* Gram-negative bacillus, *GPC* Gram-positive cocci, *KES* Klebsiella, Enterobacter, Serratia

^a^Bacteriemia was isolated in 15 (2.6%) patients

^b^At least one bacteria was identified in 406 (69.8%) patients

### Statistical analysis

Continuous variables are presented as medians with the first and third quartiles of the distributions. Categorical variables are presented as numbers and percentages. Concordance between the different scores (positive/negative) was assessed using Cohen’s Kappa coefficients and the 95% confidence intervals were computed by bootstrapping. Score positivity was compared using McNemar’s Chi-squared tests. To take into account multiple comparisons, adjusted p values were reported applying Bonferroni method. Score performances to predict mortality were assessed by computing sensitivity, specificity, positive and negative predictive values. Comparisons of specificities were realized with McNemar’s Chi-squared tests and positive predictive values were compared using relative predictive values [10]. Since there were very few missing data (between 0 and 3% depending on the variable), no specific method for handling missing data was implemented. A *p* value < 0.05 was considered statistically significant. All the analyses were realized using R software version 3.6.0. R Core Team (2019). R: a language and environment for statistical computing. R Foundation for Statistical Computing, Vienna, Austria. URL https://www.R-project.org/.

## Results

### Concordance between ISTH overt-DIC and JAAM-DIC scores for DIC diagnosis

**Fig. 1:**
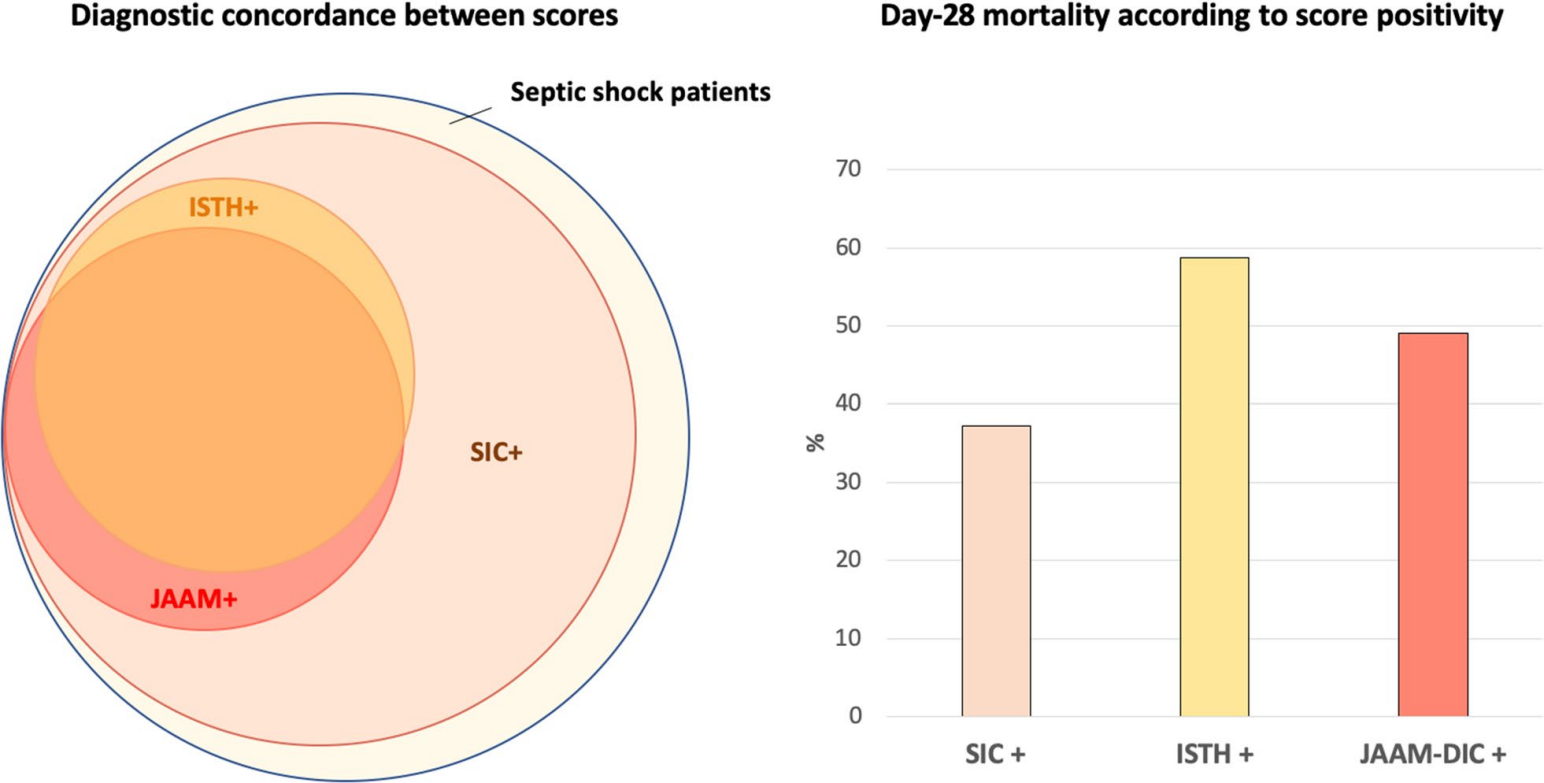
Concordance between DIC scoring systems on admission for septic shock and corresponding mortality at D28. Total SIC score is 4 or more; total JAAM-DIC score is 4 or more; total ISTH overt-DIC score is 5 or more

Markedly different DIC diagnosis rates were observed for the ISTH overt-DIC, the JAAM-DIC, and the SIC scores. Among the 582 patients, 182/567 (32.1%) were diagnosed as having a DIC on day 1 and/or on day 2 (positive score on day 1 and/or day 2) according to ISTH overt-DIC score, and 193/561 (34.4%) according to JAAM-DIC score, and 486/577 patients (84.2%) were diagnosed with a “coagulopathy” according to SIC score. These relations are shown in Fig. 1.

A moderate concordance was observed between ISTH overt-DIC and JAAM-DIC (κ = 0.67 [0.60, 0.73], *p* < 0.001). The concordance was strong for JAAM-DIC and SIC + JAAM-DIC scores (κ = 0.98 [0.96, 0.99], *p* < 0.001) and perfect between ISTH overt-DIC and SIC + ISTH overt-DIC (κ = 1).

At the time of biological diagnosis of DIC according to ISTH score, most patients (145/182 patients, 79.7%) displayed only mild or insidious clinical symptoms of consumption or even non-symptomatic disease, while thromboembolic and hemorrhagic manifestations became obvious or even life-threatening in 47.8% (87/182 patients) in the days following biological DIC diagnosis.

### Earlier SIC score positivity, but worse diagnosis specificity

To test whether SIC score positivity preceded JAAM-DIC and ISTH overt-DIC scores, we compared the rate of positive scores for the different times and classified the patients between “early DIC” (positive score at day 1, whichever was the score at day 2) and “late DIC” (negative score at day 1, and positive score at day 2). The “all DIC” group included patients with a positive score at day 1 and/or at day 2.

For the early diagnosis (“early DIC”), the rate of positive tests was not different between JAAM-DIC and ISTH overt-DIC scores (22.8% vs 21.0%, *p* = 0.60). These results were similar for late diagnosis (“late DIC”) and global diagnosis (“all DIC”) (no difference between JAAM-DIC and ISTH overt-DIC scores, 10.7% vs 10.6%, *p* = 1 and 34.4% vs 32.1%, *p* = 0.30, respectively).

For early, late and global diagnosis, SIC score was positive earlier than the two other scores (*p* < 0.001), but had worse diagnosis specificity, with 74.9% of the patients with septic shock being diagnosed with “coagulopathy” by day 1, and 84.2% by day 2 (Table 2).

**Table 2:** Number of patients on day of DIC diagnosis by the three sets of diagnostic criteria

N (%)	JAAM-DIC	SIC	ISTH overt-DIC	p-value
Early DIC	133 (22.8)	436 (74.9)	122 (21.0)	< 0.001
Late DIC	60 (10.7)	50 (8.7)	60 (10.6)	0.398
All DIC	193 (34.4)	486 (84.2)	182 (32.1)	< 0.001

Early DIC: positive score at day 1, whichever was the score at day 2; late DIC: negative score at day 1, and positive score at day 2; all DIC: positive score at day 1 and/or at day 2

### SIC score is not specific for early mortality prediction

We have then assessed the performances of the different scores for predicting early (day 7) mortality of septic shock patients. Mortality rate of the patients with a SIC score of 4 or more was lower than the one of patients with an ISTH overt-DIC score of 5 or more or a JAAM-DIC score of 4 or more (Fig. 1).

As a result, the specificity of SIC score alone to predict mortality was very low (0.18 [0.15; 0.22]) compared to the one of JAAM-DIC score (0.71 [0.67; 0.75]), *p* < 0.001, and ISTH overt-DIC score (0.76 [0.72; 0.80]), *p* < 0.001 (Table 3). The comparisons of positive predictive values (PPV) of SIC score versus JAAM-DIC, SIC score versus ISTH overt-DIC score, and JAAM-DIC score versus ISTH overt-DIC score were all < 0.001 (Table 3, Additional file 2: Table S2). Furthermore, there was no gain in sensitivity and specificity of combining SIC score to JAAM-DIC score or to ISTH overt-DIC score, compared to JAAM-DIC score alone or ISTH overt-DIC score.

**Table 3:** Performance of the different scores in predicting early mortality (day 7) of septic shock patients

Scores	Sensitivity	Specificity	PPV	NPV
SIC	0.95 [0.89; 0.98]	0.18 [0.15; 0.22]	0.22 [0.18; 0.26]	0.93 [0.86; 0.97]
JAAM-DIC	0.61 [0.50; 0.70]	0.71 [0.67; 0.75]	0.31 [0.25; 0.38]	0.89 [0.86; 0.92]
SIC + JAAM-DIC	0.61 [0.50; 0.70]	0.72 [0.68; 0.76]	0.32 [0.25; 0.39]	0.90 [0.86; 0.92]
ISTH overt-DIC	0.68 [0.58; 0.77]	0.76 [0.72; 0.80]	0.38 [0.31; 0.45]	0.91 [0.88; 0.94]
SIC + ISTH overt-DIC	0.68 [0.58; 0.77]	0.76 [0.72; 0.80]	0.38 [0.31; 0.45]	0.91 [0.88; 0.94]

*PPV* positive predictive value, *NPV* negative predictive value

## Discussion

The present study was performed in order to clarify DIC diagnosis global confusion in septic shock patients. Even if many scores are currently available, it has already been shown that they were not adequate for all diseases and when considering critically ill patients, the only score that has been validated is the JAAM 2006 one [11]. Yet, the identification of patients with septic shock-induced DIC who may benefit from anticoagulant therapy has become a major challenge in ICU [12, 13]. Indeed, if no specific treatment of DIC has proven its efficacy yet, an early diagnosis of DIC would allow the adequate stratification of septic shock patients according to their coagulation activation stage and their appropriate potential enrollment in future clinical trials, between those who might benefit from a specific treatment of coagulopathy and those who are likely not to benefit from it. Then, as we previously assessed, patients with a pre-DIC stage have the same mortality rate as those with patent DIC at ICU admission [3]. Clinical signs may also appear too late compared to the first signs of coagulation activation as shown in the present cohort to hope to improve the patients with a specific treatment, emphasizing the importance of biological scores to make early diagnosis of DIC.

Gando et al. wrote that “scoring systems are successful if clinicians can use them at the bedside, if they are readily available, and if they are easy to use” [11]. In the recommendations recently published [1], Iba et al. reviewed and highlighted the performances of SIC score to predict mortality. SIC score associates “readily available” biological values (platelets and INR) and SOFA score. SOFA score is a composite and complex clinical–biological score, including four items (respiratory SOFA, cardiovascular SOFA, hepatic SOFA, renal SOFA). It may be considered as easy to use at bedside, provided SOFA score is used as a binary score (< 2 or ≥ 2 points). In fact, as all septic shock patients have, by definition, a SOFA score greater than two points at ICU admission, this latter parameter probably does not add much in these patients. SIC would allow the detection of patients with “coagulopathy” who are at high-risk of developing DIC. SIC score was not supposed to be used alone, but combined with ISTH overt-DIC score if SIC score was positive. We have, however, shown that SIC score roughly detected all the patients (84.2%) admitted on ICU for septic shock as having “a coagulopathy”, therefore suggesting that the combination of [SIC and ISTH overt-DIC score if SIC was positive] could probably be simplified by [ISTH overt-DIC score alone]. One could indeed consider that all septic shock patients are at risk of developing DIC. Consistent with this proposal, the first validation study thus showed no advantage of SIC score over ISTH overt-DIC score in diagnosing sepsis associated DIC [14]. This impression of futility is emphasized by the perfect correlation of ISTH overt-DIC alone and its combination with SIC, as all the patients with an ISTH overt-DIC of 5 or more also had a SIC score of 4 or more, and the nearly perfect correlation of JAAM-DIC score alone and SIC combined with JAAM-DIC scores.

The JAAM 2006 score was suggested to be more sensitive than the ISTH overt-DIC score for DIC diagnosis, for example in trauma patients [15], and to allow earlier DIC diagnosis than the ISTH DIC score [5]. From a pathophysiological viewpoint, the JAAM-DIC score would be more suitable than the ISTH overt-DIC to diagnose DIC in septic patients, as it integrates the kinetics of platelet drop, higher D-dimer values and exclude fibrinogen which will remain at falsely normal or even higher levels until the late stage in acute inflammatory processes. Yet, we have shown a strong correlation between ISTH overt-DIC and JAAM-DIC scores {McHugh 2012 #2201}, suggesting that either score could be used for diagnosis of septic shock-induced DIC. When considering the delay for diagnosis, the JAAM-DIC score documented more patients as “DIC positive” than the ISTH overt-DIC one at any timepoint, suggesting that the difference between JAAM-DIC score and the ISTH overt-DIC is possibly more related to the threshold used to define DIC, as suggested by Dempfle et al. [16].

Patients diagnosed with DIC by current DIC scoring systems display an unfavorable clinical outcome, independently of the underlying disease [17]. In a first retrospective analysis of the nationwide survey for recombinant human soluble thrombomodulin, Iba et al. [18] showed that a SIC score of 4 points or more had a higher predictive value for 28-day mortality than the JAAM-DIC score. In this latter cohort, patients with a SIC score above 4 had a 28-day mortality rate of 38.4% and those with a score above 6 had a mortality rate over 45%. Our data show nearly the same mortality rate for a positive SIC score of > 4 points (37.2% at day 28), but higher VPP for ISTH overt-DIC and JAAM-DIC scores for mortality prediction.

Iba et al. [17] also showed that in septic patients with disseminated intravascular coagulation, delta SOFA, delta ISTH overt-DIC score, and delta JAAM-DIC score were correlated with 28-day mortality, with a better accuracy of delta SOFA than delta ISTH overt-DIC score (80.5 versus 66.7%, *p* < 0.001). In this study, the areas under the curve for mortality were 0.812 for delta SOFA, 0.655 for delta ISTH overt-DIC score, and 0.693 for delta JAAM-DIC score. The delta SOFA has therefore the strongest association with the 28-day mortality in patients with sepsis and DIC [17]. Yet, including points for a SOFA ≥ 2 in SIC score looks useless, as in our cohort, all septic shock patients have a SOFA ≥ 2 on ICU admission (11.0 ± 3.2 points), including patients who were included before the change of definition of sepsis and septic shock.

Our study has several strengths and some limitations. Among the strengths, we have enrolled a large and multicenter cohort of septic shock patients that were well characterized clinically. Then, data collection was prospective, therefore allowing a low rate of missing data. Finally, we have compared the main scores and their revised form, taking into account the recent recommendations for DIC diagnosis.

The main limitation of the study is that we do not have the gold standard comparator that could help assess the validity of other scores. Then we have not compared the performances of the aforementioned scores to the recently identified new diagnostic markers, like endothelial microvesicles, neutrophil fluorescence or the degree of NETs formation [3, 19–21].

Overall, our data suggest that SIC score is probably not the simple and readily available score that will revolutionize DIC diagnosis. If JAAM-DIC and ISTH overt-DIC scores seem to be both usable in septic shock patients, future research should focus on the validation of early diagnosis tools of sepsis-induced DIC [13]. In the meantime, although JAAM-DIC and ISTH overt-DIC scores may look uneasy to use due to the weighting of items, simple electronic devices may help calculate these scores daily at bedside and one should therefore consider performing these scores regularly in patients at risk of developing DIC, like in septic shock patients.

## Supplementary information

**Additional file 1: Table S1.** components of the different scoring systems.

**Additional file 2: Table S2.** AUC of continuous scores.

## Data Availability

All data generated or analyzed during this study are included in this published article.

## Abbreviations

DIC: Disseminated intravascular coagulation
ICU: Intensive care unit
INR: International Normalized Ratio
ISTH: International Society on Thrombosis and Haemostasis
JAAM: Japanese Association for Acute Medicine
PT: Prothrombin time
SAPS II: Simplified acute physiology score II
SIC: Sepsis-induced coagulopathy
SOFA: Sequential organ failure assessment
