# Supplementary material for: Performances of disseminated intravascular coagulation scoring systems in septic shock patients
Source: Ann Intensive Care. 2020 Jul 10;10:92. doi: 10.1186/s13613-020-00704-5 (PMC7352012; doi:10.1186/s13613-020-00704-5)
Supplement: Supplementary file 1 — Additional file 1: Table S1. components of the different scoring systems. [file 13613_2020_704_MOESM1_ESM.docx]

**Additional file 1: Table S1:** components of the different scoring systems

| **Parameters** | **ISTH Score** | **JAAM-DIC 2016 Score** | **SIC Score** |
| --- | --- | --- | --- |
| **Antithrombin (%)** | - | [0] ≥ 70  [1] < 70 | - |
| **Platelets (G/L)** | [0] ≥ 100  [1] 50-99  [2] < 50 | [0] ≥ 120  [1] 80-119 or ≥30% decrease in 24hrs  [3] < 80 or ≥ 50% decrease in 24hrs | [0] ≥ 150  [1] 100-149  [2] < 100 |
| **PT (%) / INR** | PT  [0] ≥ 64  [1] 35-63  [2] < 35 | PT  [0] ≥ 64  [1] < 63 | INR  [0] <1.2  [1] 1.2-1.4  [2] > 1.4 |
| **Fibrinogen (g/L)** | [0] ≥ 1.0  [1] < 1.0 | - | - |
| **D-dimers (μg/mL)** | [0] ≤ 0.5  [2] moderate increase  [3] strong increase | [0] ≤ 5.0  [1] 5.0-15.0  [3] > 15.0 | - |
| **SOFA score (points)** | - | - | [0] <1  [1] 1  [2] ≥ 2 |
| **Positive score** | **≥ 5/8 points** | **≥ 4/8 points** | **≥ 4/6 points** |

INR: International Normalized Ratio; PT: prothrombin time; SIC: sepsis-induced coagulopathy; SOFA: Sepsis-related Organ Failure Assessment
