# Supplementary material for: Performances of disseminated intravascular coagulation scoring systems in septic shock patients
Source: Ann Intensive Care. 2020 Jul 10;10:92. doi: 10.1186/s13613-020-00704-5 (PMC7352012; doi:10.1186/s13613-020-00704-5)
Supplement: Supplementary file 2 — Additional file 2: Table S2. AUC of continuous scores. [file 13613_2020_704_MOESM2_ESM.docx]

**Additional file 2: Table S2: AUC of continuous scores**

| **Scores** | **AUC** |
| --- | --- |
| **SIC** | 63.2 [57.8; 68.7] |
| **JAAM-DIC** | 66.2 [60.6; 71.8] |
| **ISTH overt-DIC** | 69.3 [63.6; 75.1] |

P=0.24 between JAAM-DIC and ISTH overt DIC scores;

P=0.33 between JAAM-DIC and SIC scores;

P=0.01 between ISTH overt DIC and SIC scores.
